# Complete Genome Sequence of *Alkaliphilus metalliredigens* Strain QYMF, an Alkaliphilic and Metal-Reducing Bacterium Isolated from Borax-Contaminated Leachate Ponds

**DOI:** 10.1128/genomeA.01226-16

**Published:** 2016-11-03

**Authors:** C. Hwang, A. Copeland, S. Lucas, A. Lapidus, K. Barry, J. C. Detter, T. Glavina del Rio, N. Hammon, S. Israni, E. Dalin, H. Tice, S. Pitluck, O. Chertkov, T. Brettin, D. Bruce, C. Han, J. Schmutz, F. Larimer, M. L. Land, L. Hauser, N. Kyrpides, N. Mikhailova, Q. Ye, J. Zhou, P. Richardson, M. W. Fields

**Affiliations:** a Center for Biofilm Engineering, Montana State University, Bozeman, Montana, USA; b DOE Joint Genome Institute, Walnut Creek, California, USA; c State Key Laboratory of Estuarine and Coastal Research, East China Normal University, Shanghai, China; d University of Oklahoma, Department of Microbiology and Plant Biology, Norman, Oklahoma, USA; e Department of Microbiology and Immunology, Montana State University, Bozeman, Montana, USA

## Abstract

*Alkaliphilus metalliredigens* strain QYMF is an anaerobic, alkaliphilic, and metal-reducing bacterium associated with phylum *Firmicutes*. QYMF was isolated from alkaline borax leachate ponds. The genome sequence will help elucidate the role of metal-reducing microorganisms under alkaline environments, a capability that is not commonly observed in metal respiring-microorganisms.

## GENOME ANNOUNCEMENT

*Alkaliphilus metalliredigens* QYMF is a strict anaerobic bacterium isolated from alkaline borax leachate ponds with concentrations of sodium and boron that ranged from 0.04 to 0.53 M and 0.19 and 0.28 M, respectively, at U. S. Borax Company (Boron, CA). QYMF is capable of utilizing Fe(III)-citrate, Fe(III)-EDTA, Co(III)-EDTA, and Cr(VI) as electron acceptors during growth with yeast extract or lactate as electron donors. Optimum growth conditions for QYMF with the metals listed above were observed at pH 9.6, 35°C, and a NaCl concentration of 20 g/L in the presence of 2 g/L of borate (1). The metal-reducing capability under alkaliphilic growth conditions (up to pH 11) in the presence of elevated salt levels has not been demonstrated in other *Alkaliphilus* isolates (2–6) nor other metal-reducing bacteria. The novel and unique lifestyle of QYMF warranted the determination of the genome sequence as it pertains to a better appreciation of the genetic and physiological diversity of metal-reducing microorganisms that thrive in extreme environmental conditions, including toxic metal-contaminated sites. Moreover, the genomic data provides insight into the biogeochemical processes on early Earth, which can lend knowledge to the exploration of the boundaries for extraterrestrial microbial life.

The genome sequence was determined with the Sanger sequencing method by the U.S. DOE Joint Genome Institute (JGI). The completed genome was curated and annotated as previously described (7–9). Putative gene functions were also assigned with public databases including COG, KEGG, Pfam, TIGRFam, and InterPro. The resultant genome size is 4.93 Mb with 36.8% G+C content. Genome sequences of three other *Alkaliphilus* species have been completed. While the G+C content is comparable to other species, QYMF has a larger genome size than *A. oremalandii* strain OhILAs (3.12 Mb, 36.3% G+C), *A. transvaalensis* (4.02 Mb, 33.95% G+C), and *A. peptidifermentans* (4.45 Mb, 34.09% G+C). Comparison of SSU rRNA gene shows that QYMF has 93 to 96% nucleotide identity to these sequenced *Alkaliphilus* species. The genome sequence of QYMF contains 5,016 identified putative genes, of which 4,801 are protein coding genes with functions predicted for 3,130 genes, a total of 31 rRNA genes (11 are 5S rRNA gene and 10 are 16S and 23S rRNA gene each), 106 tRNA genes, and 78 other RNA genes.

Although growth of QYMF with arsenic has not been characterized, the leachate ponds from where QYMF was isolated contained As concentrations of approximately 1.7 mM. Genome analysis of QYMF indicates the presence of genes encoding arsenical resistance proteins and two novel *ars* operons that encodes arsenite efflux permeases (Acr3), whose mechanism of transport was characterized by Fu et al. (10, 11). Recently, the *aro*A gene of QYMF, which encodes for the enzyme 5-enopyruvylshikimate-3-phosphate synthase, was shown to have the potential in developing glyphosate-resistant crops (12). This implicates the potential use of arsenite efflux mechanism of QYMF as a strategy to reduce As accumulation in rice, which has been demonstrated with the expression of ScAcr3p, an arsenite plasma membrane transporter, from *Saccharomyces cerevisiae* to rice grains (13). Future work should assess the use of metal-tolerant bacteria in various applications.

### Accession number(s).

*A. metalliredigens* was deposited at GenBank, National Center for Biotechnology Information, under accession number CP000724.
